# Identification of novel molecular signatures of IgA nephropathy through an integrative -omics analysis

**DOI:** 10.1038/s41598-017-09393-w

**Published:** 2017-08-22

**Authors:** Magdalena Krochmal, Katryna Cisek, Szymon Filip, Katerina Markoska, Clare Orange, Jerome Zoidakis, Chara Gakiopoulou, Goce Spasovski, Harald Mischak, Christian Delles, Antonia Vlahou, Joachim Jankowski

**Affiliations:** 1 Biomedical Research Foundation Academy of Athens, Center of Basic Research, Athens, Greece; 2 RWTH Aachen University Hospital, Institute for Molecular Cardiovascular Research, Aachen, Germany; 3 Mosaiques Diagnostics GmbH, Hannover, Germany; 4 University of Maastricht, CARIM School for Cardiovascular Diseases, Maastricht, Netherlands; 5 University of Glasgow, Institute of Cardiovascular and Medical Sciences, Glasgow, UK; 6 Institute of Cardiovascular and Medical Sciences, BHF Glasgow Cardiovascular Research Centre, University of Glasgow, 126 University Place, Glasgow, G12 8TA UK; 7 Department of Pathology, School of Medicine, University of Glasgow, Glasgow, UK; 8 Department of Nephrology, Medical Faculty, University of Skopje, Skopje, Macedonia; 9 Pathology Department, National and Kapodistrian University of Athens, Athens, Greece

## Abstract

IgA nephropathy (IgAN) is the most prevalent among primary glomerular diseases worldwide. Although our understanding of IgAN has advanced significantly, its underlying biology and potential drug targets are still unexplored. We investigated a combinatorial approach for the analysis of IgAN-relevant -omics data, aiming at identification of novel molecular signatures of the disease. Nine published urinary proteomics datasets were collected and the reported differentially expressed proteins in IgAN vs. healthy controls were integrated into known biological pathways. Proteins participating in these pathways were subjected to multi-step assessment, including investigation of IgAN transcriptomics datasets (Nephroseq database), their reported protein-protein interactions (STRING database), kidney tissue expression (Human Protein Atlas) and literature mining. Through this process, from an initial dataset of 232 proteins significantly associated with IgAN, 20 pathways were predicted, yielding 657 proteins for further analysis. Step-wise evaluation highlighted 20 proteins of possibly high relevance to IgAN and/or kidney disease. Experimental validation of 3 predicted relevant proteins, adenylyl cyclase-associated protein 1 (CAP1), SHC-transforming protein 1 (SHC1) and prolylcarboxypeptidase (PRCP) was performed by immunostaining of human kidney sections. Collectively, this study presents an integrative procedure for -omics data exploitation, giving rise to biologically relevant results.

## Introduction

IgA nephropathy (IgAN) is the most common form of primary glomerular disease worldwide, progressing to renal failure in almost one third of cases^1, 2^. IgAN is characterized by the presence of mesangial deposits of IgA-containing immune complexes. Clinical manifestation of the disease is highly variable with symptoms ranging from microscopic to severe proteinuria, haematuria and histopathological lesions that might include mild mesangial thickening to membranoproliferative glomerulonephritis^3^. Moreover, IgAN prevalence depends on ethnicity, with highest in Asians and lowest amongst African-Americans^4^ and has unequal gender distribution, affecting males more frequently than females (male-to-female ratio of 3:1 in Europeans)^5^. Currently, renal biopsy remains the gold standard of diagnosis of IgAN; however, due to its invasive nature, it is routinely not performed at often asymptomatic, early stages or for serial monitoring of disease progression^6, 7^.

The pathogenesis of IgAN currently remains only partially understood, despite numerous studies that helped in elucidation of several molecular mechanisms involved in the disease. These collectively resulted in the development of the IgAN autoimmune pathogenesis model, proposing disease development in a four-hit manner: (1) overproduction of aberrantly glycosylated IgA1; (2) production of glycan-specific IgG and IgA autoantibodies recognizing the undergalactosylated IgA1 molecule; (3) triggering the formation of immune complexes; (4) and their accumulation in the glomerular mesangium, which initiates renal injury through mesangial cells and complement activation^8, 9^. Nevertheless, many questions stay unanswered, such as the cause of preferential deposition of immune complexes in mesangium and its connection with proteinuria and haematuria.

According to clinical practice guidelines for IgAN treatment^10^, proteinuria is considered the strongest prognostic factor in IgAN. Recommended treatment options aim at slowing the disease progression and are limited to blood pressure control or non-specific immunosuppression with corticosteroids or other immunosuppressive agents^11, 12^. Currently, disease-specific therapy does not exist, however, on-going clinical trials focus on assessing both traditional treatments and emerging therapies^11^. In brief, benefit of immunosuppressive treatment in standard care is being evaluated in the TESTING^13^ clinical trial. Spurred by increasing evidence of excessive immunoproteasomal activity in IgAN, the “BORTEZOMIB IN IgAN” clinical trial is investigating the benefits of proteasome inhibition. Owing to a better understanding of the role of mucosal immunity, B-cell activation and mesangial cell activation due to an exaggerated immune response in IgAN became the target of new promising therapies, investigated in phase II/III clinical trials^14^. Targeting mucosal B-cell induction (NEFIGAN trial)^15^ as well as B-cell maturation and survival (BRIGHT-SC)^16^ has already shown promising preliminary results. Nonetheless, recently finalized STOP-IgAN trial^17^ assessing the added value of immunosuppression in IgAN treatment reported no additional benefit to basic supportive care^18^. Along the same lines, B-cell depletion by rituximab (RITUXIMAB IN IgAN study) showed a lack of benefit in improving kidney function^19^. Given that the efficacy of novel therapies in immune-mediated kidney diseases, including IgAN, is in most part disappointing, there is a continuous demand for better treatment strategies and novel targets^20^.

Until now, several IgAN biomarkers have been identified and validated. Serum levels of circulating galactose-deficient IgA1^21^, IgA1-containing circulating immune complexes^22, 23^ and IgG autoantibodies specific for Gd-IgA1^23^ were found to be good diagnostic biomarkers for IgAN. Additionally, serum levels of complement proteins, such as C3, factor H and fragments of complement activation proteins were found reflective of disease severity or clinical presentation^24^. Some promising biomarker candidates were also found in urine of IgAN patients, such as mannose-binding lectin (MBL) predicting disease progression^25^ or podocalyxin associated with IgAN activity^26^. Moreover, urinary peptidomics approaches have been utilized to develop non-invasive diagnostic tools based on a panel of differentially expressed molecules, able to differentiate IgAN from patients with other glomerular diseases^27^. None of the aforementioned markers has been implemented in clinical settings until now, stressing the unmet need for novel biomarkers, able to successfully diagnose early stages of the disease^28^.

Public availability of experimental data from multiple studies sparked efforts towards data integration strategies, aiming at a better understanding of complex biological systems and phenomena^29^. Information-rich –omics datasets may increase reliability and generalizability of results, and most importantly, enable generation of novel hypotheses and insights^30^. System-level analyses have gained popularity, as reflected by reported studies successfully utilizing data integration strategies^31–33^. Several different approaches and integration tools have been proposed for meta-analysis of data, however, translation of accumulated data into a meaningful biological context remains a challenge^34^. Collectively, systems biology-driven modelling pipelines are still under development and a standard, comprehensive workflow has not been established^35^.

In this study, we hypothesized that through meta-analysis of consolidated data from -omics studies on IgAN, novel biological traits relevant to the disease might be identified. Therefore, we developed a step-wise approach involving integration and pathway analysis of urine proteomics datasets, cross-validation of predictions through transcriptomics and tissue expression databases, shortlisting of candidates through further literature mining and experimental verification of selected targets by immunohistochemistry.

## Results

### Pathway analysis and functional candidates selection

**Figure 1:**
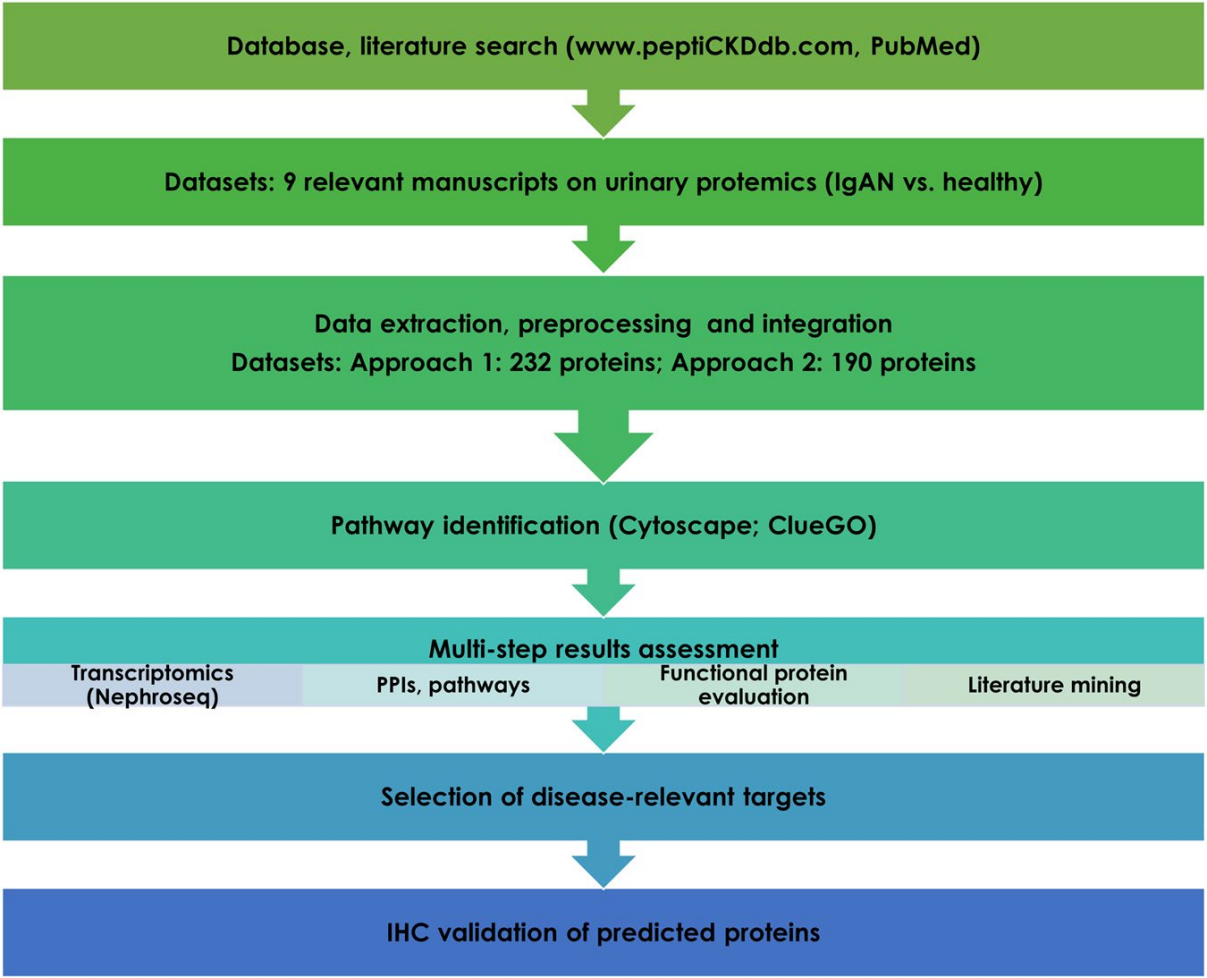
Schematic representation of the steps followed in the project. Initially, literature and database mining was performed to identify relevant datasets from IgAN human –omics experiments. Datasets were extracted from selected resources and pre-processed forming the input protein set subjected to pathway and interactome analysis. Investigation of pathways and multi-step shortlisting of predicted proteins supported by functional protein evaluation and literature mining yielded a list of disease-relevant targets, further validated in the kidney tissue via immunohistochemistry (IHC).

The overall workflow of the analysis is depicted in Fig. 1. Database search using the database peptiCKDdb^36^ for IgAN-related datasets yielded two manuscripts focusing on peptidomics analyses (sample types: urine (1), blood (1)) and 10 manuscripts focusing on proteomic analyses using urine (9), blood (1), kidney (1) as sample material. Complementary literature search provided a limited number of additional manuscripts: we identified no additional peptidomics publications, one manuscript describing proteomics (not accessible), one urinary metabolomics and five on transcriptomics analysis (profiling of leukocytes (1), blood (2) and kidney (2)). Studies of the urine proteome (9 relevant manuscripts listed in Table 1) were the most prominent, hence analysis focused on these datasets. Data corresponding to differentially expressed proteins between patients suffering from IgAN and healthy controls, as reported by the authors (with fold change values and/or regulation reported; description of the statistical approach used in each case is provided in Supplementary Table S1), were integrated resulting in a list of 236 non-redundant proteins (Supplementary Table S1). Four proteins, collagen alpha-1(VI) chain, cystatin-C, dipeptidyl peptidase 4 and uromodulin were removed from the analysis due to conflicting regulation trends in different studies. Therefore, the final analysis input consisted of 232 proteins (167 downregulated, 65 upregulated) (Supplementary Table S2).

**Table 1:** Characteristics of the urinary proteomics datasets used for the analysis. Proteomics method – mass spectrometry-based method applied in the study; Dataset size – differentially expressed proteins reported in the publication; Cohort – Total number of cases and controls).

Authors	Title	Proteomics method	Dataset size	Origin of proteins	Cohort (cases+control)
Park M.R. *et al*.^80^	Establishment of a 2-D human urinary proteomic map in IgA nephropathy.	MALDI-TOF MS	**59 proteins**	Soluble fraction	25
Rocchetti M.T. *et al*.^81^	Urine protein profile of IgA nephropathy patients may predict the response to ACE-inhibitor therapy.	nano-HPLC-ESI-MS/MS	**8 proteins**	Soluble fraction	38
Moon P.G. *et al*.^82^	Proteomic analysis of urinary exosomes from patients of early IgA nephropathy and thin basement membrane nephropathy.	LC-MS/MS	**124 proteins**	Urinary exosomes	12
Graterol F. *et al*.^83^	Poor histological lesions in IgA nephropathy may be reflected in blood and urine peptide profiling.	MALDI-TOF MS	**3 proteins**	Soluble fraction	33
Samavat S. *et al*.^84^	Diagnostic urinary proteome profile for immunoglobulin a nephropathy.	nLC-MS/MS	**46 proteins**	Soluble fraction	21
Rocchetti M.T. *et al*.^85^	Association of urinary laminin G-like 3 and free K light chains with disease activity and histological injury in IgA nephropathy.	MALDI-TOF-MS/MS	**2 proteins**	Soluble fraction	89
Yokota H. *et al*.^86^	Absence of increased alpha1-microglobulin in IgA nephropathy proteinuria.	LC-MS/MS	**10 proteins**	Soluble fraction	27
Surin B. *et al*.^87^	LG3 fragment of endorepellin is a possible biomarker of severity in IgA nephropathy.	MALDI-TOF/TOF-MS	**4 proteins**	Soluble fraction	73
Mucha K. et al.^88^	Complement components, proteolysis-related, and cell communication-related proteins detected in urine proteomics are associated with IgA nephropathy.	IEF/LC-MS/MS	**18 proteins**	Soluble fraction	60

Pathway analysis was performed via two approaches, using the full urinary proteomics dataset (Approach 1; Supplementary Table S2) and excluding bona fide plasma proteins (Approach 2; Supplementary Table S3), as these may reflect the failing glomerular filtration barrier, hence be an effect rather than the cause of the disease. Pathway enrichment of the full protein dataset yielded a list of 28 pathways (p-value < 0.05, Supplementary Table S4, Approach 1). Based on further critical evaluation, 13 pathways most likely irrelevant to IgAN) were not considered for further investigation. These included budding and maturation of HIV virion, visual phototransduction and glycolysis; marked in grey in the Supplementary Table S4. Fifteen pathways remained as potentially relevant to the studied pathology including platelet activation, signalling and aggregation, membrane trafficking, binding and uptake of ligands by scavenger receptors, metabolism of angiotensinogen to angiotensin peptides and complement cascades (Supplementary Table S4).

In comparison, after exclusion of a total of 42 plasma proteins from the analysis input the remaining 190 proteins (152 downregulated, 38 upregulated, Supplementary Table S3) yielded 19 significant pathways (p-value < 0.05, Supplementary Table S5, Approach 2). After removing six irrelevant pathways (e.g. budding and maturation of HIV virion, glucose metabolism) 13 entries remained. There was a substantial overlap (40%) between the predicted pathways in the two approaches (including/excluding plasma proteins). Specifically, pathways related to platelet activation, signalling and aggregation, membrane trafficking, and metabolism of angiotensinogen to angiotensins appeared significant in both analyses. Nevertheless, some differences could also be observed - after exclusion of plasma proteins, new pathways potentially related to tissue-level events i.e. collagen formation, collagen biosynthesis and modifying enzymes, integrin cell surface interactions and EPH-ephrin signalling, were predicted as significantly deregulated in disease.

Taking into account both analytical approaches, 20 molecular pathways were predicted to be de-regulated in IgAN (Table 2). These pathways involved 70 proteins from the input dataset and additional 657 proteins participating in these pathways. The latter were further analysed for their relevance to IgAN through a step-wise approach (summarized in Fig. 2 and described in detail below). Of note, pathway analysis of the individual proteomics datasets gave results for 5 out of 9 sets (due to the low number of protein identifications reported in the remaining 4 datasets). Moreover, comparison of the pathways yielded from the integrated and single-set analysis showed that several significant pathways do not appear in each individual analysis (e.g. platelet activation, signalling and aggregation) or appear only upon integration (e.g. complement cascade) (Supplementary Table S9).

**Table 2:** Significant pathways yielded from the analysis of full urine proteomics dataset (Approach-1) and after exclusion of plasma proteins (Approach-2). Pathways specific for Approach-1 are bolded and pathways specific for Approach-2 are underlined. The original lists of pathways obtained from both analyses can be found in the supplementary material.

#	Pathway	#genes	coverage %	p-value	Genes up-regulated	Genes down-regulated
1	Platelet activation, signalling and aggregation	23	10.45	2.55E-09	A2M, ALB, APOA1, F2, PSAP, SERPINA1, SERPINF2, TF	CFL1, EGF, FN1, GNA11, GNAI1, GNAI2, GNB1, GNB2, IGF2, KNG1, MAPK3, PFN1, PIK3R3, RAP1A, SERPING1
2	Platelet degranulation	14	17.72	2.04E-08	A2M, ALB, APOA1, PSAP, SERPINA1, SERPINF2, TF	CFL1, EGF, FN1, IGF2, KNG1, PFN1, SERPING1
3	Response to elevated platelet cytosolic Ca^2+^	14	16.67	4.78E-08	A2M, ALB, APOA1, PSAP, SERPINA1, SERPINF2, TF	CFL1, EGF, FN1, IGF2, KNG1, PFN1, SERPING1
4	**Scavenging of heme from plasma**	6	50.00	3.00E-06	ALB, APOA1, HBA2, HBB, HP	AMBP
5	Vesicle-mediated transport	19	7.95	1.31E-05	ALB, APOA1, CLTCL1, HBA2, HBB, HP	AMBP, CHMP4B, CHMP5, MYH8, RAB10, RAB14, RAB5C, TSG101, VPS37B, VPS4B, VTA1, YWHAG, YWHAQ
6	**Intrinsic Pathway of Fibrin Clot Formation**	6	27.27	2.05E-04	A2M, F2, SERPINC1	KNG1, SERPINA5, SERPING1
7	Endosomal Sorting Complex Required For Transport (ESCRT)	6	20.69	1.17E-03	—	CHMP4B, CHMP5, TSG101, VPS37B, VPS4B, VTA1
8	**Formation of Fibrin Clot (Clotting Cascade)**	6	15.38	6.81E-03	A2M, F2, SERPINC1	KNG1, SERPINA5, SERPING1
9	**Binding and Uptake of Ligands by Scavenger Receptors**	6	15.00	7.88E-03	ALB, APOA1, HBA2, HBB, HP	AMBP
10	Membrane Trafficking	13	6.47	1.06E-02	CLTCL1	CHMP4B, CHMP5, MYH8, RAB10, RAB14, RAB5C, TSG101, VPS37B, VPS4B, VTA1, YWHAG, YWHAQ
11	Metabolism of Angiotensinogen to Angiotensins	4	25.00	1.47E-02	—	ACE, ANPEP, ENPEP, MME
12	ADP signalling through P2Y purinoceptor 12	4	18.18	2.25E-02	—	GNAI1, GNAI2, GNB1, GNB2
13	Signal amplification	5	15.63	2.45E-02	—	GNA11, GNAI1, GNAI2, GNB1, GNB2
14	**Thrombin signalling through proteinase activated receptors (PARs)**	5	15.63	2.45E-02	F2	GNA11, GNB1, GNB2, MAPK3
15	Collagen formation	7	8.14	2.75E-02	COL11A1, COL11A2, COL15A1, COL17A1, COL22A1, COL4A4	CTSB
16	Collagen biosynthesis and modifying enzymes	6	9.38	3.31E-02	COL11A1, COL11A2, COL15A1, COL17A1, COL22A1, COL4A4	
17	Integrin cell surface interactions	6	9.09	3.91E-02	COL4A4	AGRN, CD44, CDH1, FN1, HSPG2
18	EPH-Ephrin signalling	7	7.53	4.42E-02	ACTB, CLTCL1	ACTG1, ARHGEF7, CFL1, MYH9, SDCBP
19	**Regulation of Insulin-like Growth Factor (IGF) transport and uptake by Insulin-like Growth Factor Binding Proteins (IGFBPs)**	4	19.05	4.49E-02	F2	IGF2, IGFALS, KLK1
20	**Complement cascade**	5	13.51	4.90E-02	C3, C4A	C7, CD55, MASP2

**Figure 2:**
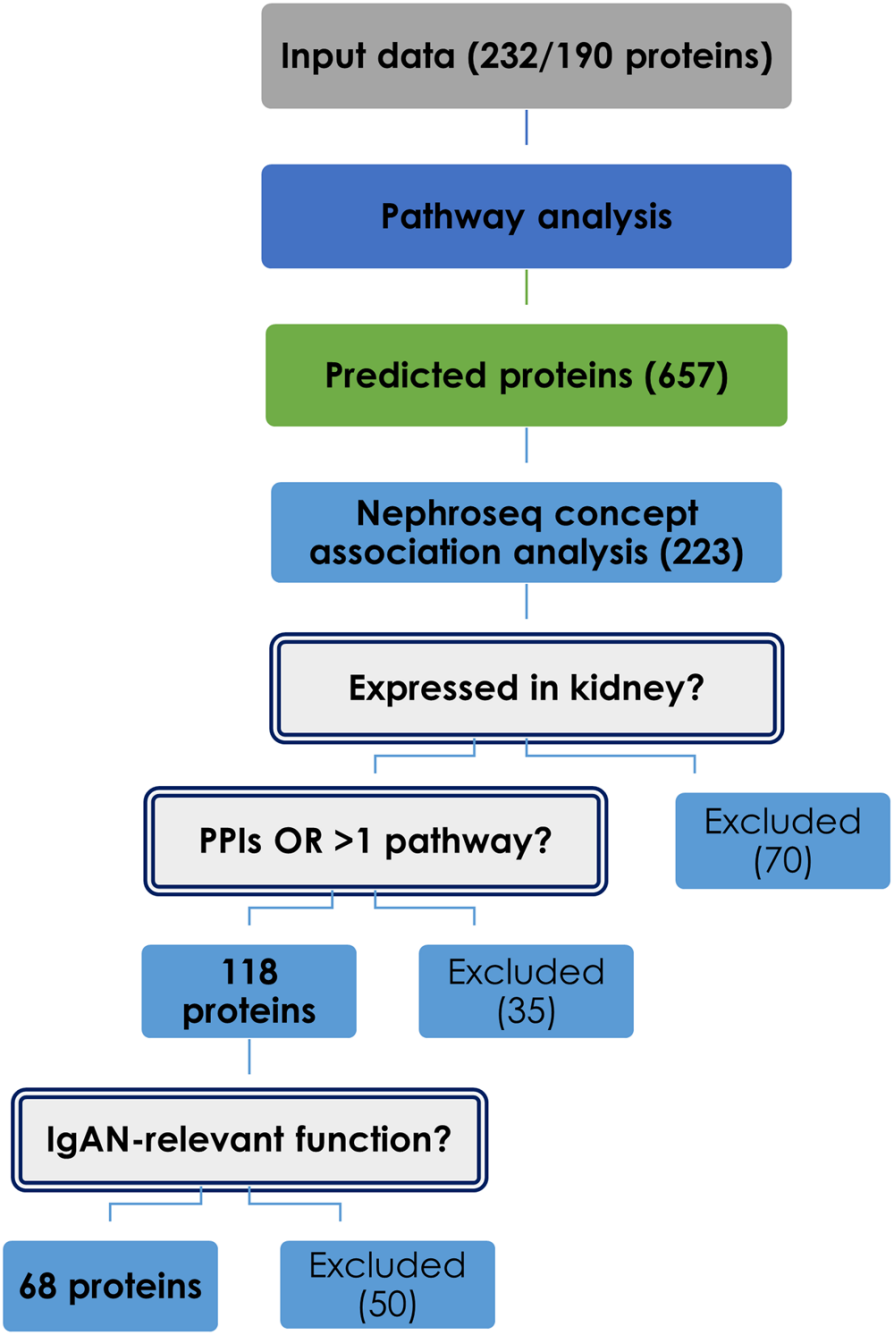
Classification tree used for selection of validation candidates based on the results from Cytoscape pathway analysis. The integrated dataset constructed by combining data from 9 urine proteomics manuscripts was subjected to pathway analysis in Cytoscape (ClueGO plug-in). 20 pathways were found significant. Predicted molecules involved in the pathways were evaluated in order to identify novel molecules potentially involved in IgAN pathology. The process of multi-step assessment included transcriptomics association analysis (Nephroseq database) and investigation of tissue expression data (Human Protein Atlas), followed by application of protein-protein interactions (STRING database) and pathway occurrence thresholds, as well as functional evaluation (UniProt, GeneOntology). 68 shortlisted molecules were subjected to detailed literature mining in order to select the most disease-relevant findings.

### Nephroseq concept association analysis

Nephro﻿seq asso﻿ci﻿ation analysis was performed to identify common molecular features between the predicted 657 pathway building proteins and a collection of gene expression signatures related to IgAN i.e. Nephroseq “concepts”. A total of five IgAN-derived concepts were identified in the analysis (respective demographics and clinical information are summarized in Supplementary Table S6). Jointly, 223 IgAN-associated transcripts from Nephroseq were found overlapping with the 657 proteins from the input data (Supplementary Table S7). Those were subjected to further steps of assessment.

### Tissue expression, PPIs, pathway count

Following the concept analysis in Nephroseq, the 223 shortlisted proteins were further examined using the Human Protein Atlas database for their expression in healthy kidney tissue. A subset of 153 proteins was reported to be expressed in kidney tissue. These were shortlisted based on the number of corresponding pathways as well as their potentially central role in these pathways, as reflected by number of their protein-protein interactions. Based on the latter analysis, a subset of 118 proteins forming more than one protein-protein interaction (STRING database) and/or being involved in more than one identified pathway were selected for further functional assessment. The individual proteins with corresponding assessment are presented in Supplementary Table S7.

### Assessment of function

**Figure 3:**
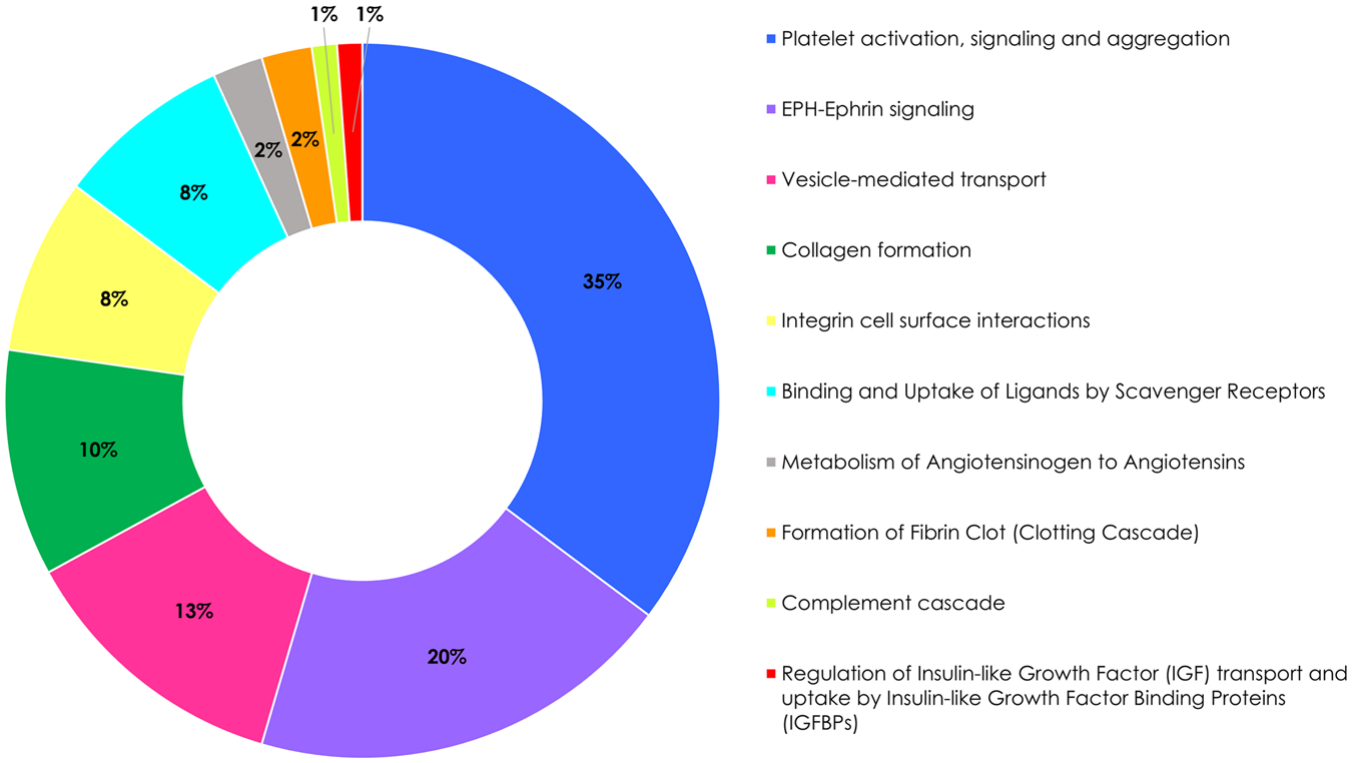
Distribution of 68 shortlisted proteins among pathways yielded from the pathway enrichment analysis.

The 118 proteins remaining after the aforementioned shortlisting steps were screened for their potential function in IgAN-related processes, namely function in immunity/autoimmunity, blood pressure regulation, vascular injury, oxidative stress and ECM remodelling. An initial screening was performed by using UniProt and Gene Ontology databases, which highlighted 68 proteins of potentially higher relevance (Supplementary Table S8). The majority of shortlisted molecules (35%) originated from the statistically most significant pathway, platelet activation, signalling and aggregation pathway followed by EPH-ephrin signalling (20%), vesicle mediated transport (13%), collagen formation (10%), integrin cell surface interactions (8%) and binding and uptake of ligands by scavenger receptors (8%) pathways (Fig. 3).

Consequently, literature databases were examined to place these findings in the context of existing IgAN literature and reveal novel findings. Individual assessment of shortlisted proteins resulted in a list of the 20 most significant proteins predicted in the analysis, summarized in Table 3. Several proteins were previously described in the context of different renal diseases or relevant animal models as indicated in the table, including six molecules reported to be differentially expressed in the kidney tissue of patients suffering from IgAN, namely ACTN1^37^, ACTN4^38^, GAS6^39^, PRR^40^, SPARC^41^ and MMP-2^42^, supporting the validity of our approach.

**Table 3:** Summary of the most significant findings predicted following application of a series of steps involving pathway analysis, as well as cross-checking through transcriptomics (Nephroseq concept association analysis), protein expression (Human Protein Atlas) and protein-protein interactions (STRING) databases and literature mining.

Gene	Protein name	Function (UniProt)	Literature
**Platelet activation, signalling and aggregation**			
**ACTN1**	Alpha-actinin-1	actin anchoring, bundling protein	Validated in IgAN kidney tissue^37^
**ACTN4**	Alpha-actinin-4	actin anchoring, bundling protein	Validated in IgAN kidney tissue^38^
**CAP1**	Adenylyl cyclase-associated protein 1	complex developmental and morphological processes, actin cytoskeleton organization	functional receptor for human resistin, inflammatory action of monocytes^60^
**GAS6**	Growth arrest-specific protein 6	ligand, negative regulation of renal albumin absorption, cell growth and survival, implicated in hypertension	Validated in IgAN kidney tissue^39^
**GNAQ**	Guanine Guanine nucleotide-binding protein G(q) subunit alpha	modulators or transducers in various transmembrane signalling systems, modulation of B-cell selection and survival	maintenance of B-cell tolerance, autoimmunity repression^89^
**PTPN1**	Tyrosine-protein phosphatase non-receptor type 1	Tyrosine-protein phosphatase, regulator of endoplasmic reticulum unfolded protein response	control of B cell activation and the maintenance of immunological tolerance, induction of migratory podocyte response^90–92^
**SHC1**	SHC-transforming protein 1	Signalling adapter, couples activated growth factor receptors to signalling pathways	pivotal regulator of oxidative stress (p66Shc isoform), putative marker of tubular oxidative injury in DN^71^
**WDR1**	WD repeat-containing protein 1	disassembly of actin filaments	platelet-related complications in CKD^93^
**EPH-Ephrin signalling**			
**ADAM10**	Disintegrin and metalloproteinase domain-containing protein 10	protease	expressed in human podocytes, may be involved in the development of glomerular kidney diseases through Notch signalling pathway^94^
**EFNA4**	Ephrin-A4	ligand for Eph receptors, interaction between activated B-lymphocytes and dendritic cells in tonsils	signalling in injury and inflammation^95^
**Collagen formation**			
**BMP1**	Bone morphogenetic protein 1	protease	involved in renal fibrosis^96^
**CTSS**	Cathepsin S	thiol protease	promotes vascular inflammation and calcification^97, 98^
**PLOD2**	Procollagen-lysine,2-oxoglutarate 5-dioxygenase 2	Collagen crosslinking enzyme	—
**Integrin cell surface interactions**			
**F11R**	Junctional adhesion molecule A	epithelial tight junction formation, regulation of monocyte transmigration	possible pathogenic role in arterial hypertension^99^
**JAM3**	Junctional adhesion molecule C	cell-cell adhesion, mediator of angiogenesis, regulation of transepithelial migration of polymorphonuclear neutrophils	inflammation and vascular biology, mediation of leukocyte infiltration in response to ischemia reperfusion injury^100^
**Binding and Uptake of Ligands by Scavenger Receptors**			
**SPARC**	SPARC	regulation of cell growth through interactions with the extracellular matrix and cytokines	Validated in IgAN kidney tissue^41^
**STAB1**	Stabilin-1	scavenger receptor for acetylated low density lipoprotein	Maintenance of tissue homeostasis^68, 69^
**Metabolism of Angiotensinogen to Angiotensins**			
**ATP6AP2/PRR**	Renin receptor	renin and prorenin cellular receptor, may play a role in the renin-angiotensin system (RAS)	Validated in IgAN kidney tissue^40^
**Formation of Fibrin Clot (Clotting Cascade)**			
**PRCP**	Prolylcarboxypeptidase	peptidase	activity associated with cardiovascular risk factors, such as atherosclerosis, inflammation, and diabetes^75^
**Regulation of Insulin-like Growth Factor (IGF) transport and uptake by Insulin-like Growth Factor Binding Proteins (IGFBPs)**			
**MMP2**	Matrix metalloproteinase-2	protease, remodelling of the vasculature, angiogenesis, tissue repair, tumour invasion, inflammation, and atherosclerotic plaque rupture	Validated in IgAN kidney tissue^42^

To further investigate the reliability of our method, three proteins predicted to be functionally relevant in IgAN were selected for assessment of their expression in the kidney tissue via immunohistochemistry (IHC). Moreover, selection of those targets was guided by their novelty in the context of IgAN and availability of specific antibodies. Based on the bioinformatics analysis, we hypothesized that these proteins, adenylyl cyclase-associated protein 1 (CAP1), SHC-transforming protein 1 (SHC1) and prolylcarboxypeptidase (PRCP), are likely upregulated in the kidney tissue of IgAN patients compared to healthy controls.

### Validation of selected targets

Immunohistochemistry staining for validation targets, CAP1, SHC1 and PRCP, was performed on eight IgAN, three other glomerular diseases and one healthy control slides of kidney tissue. No staining was observed in case of negative controls, confirming the specificity of the IHC analysis. Clinical information, intensity levels (individual IHC scores) and respective images can be found in Table 4, Supplementary Table S10 and Supplementary Fig. S1 and S2, respectively. CAP1 showed negative/below limit of detection (−) staining in the normal kidney, negative (−) to strong (+++) staining in other glomerular diseases and weak (+) to strong (+++) staining in tubules and glomeruli with noticeable staining in inflammatory cells in the IgAN group. SHC1 and PRCP presented weak (+) staining in control kidney tissue and weak (+) to strong (+++) staining in the samples of other glomerular diseases and in the IgAN group. Overall, staining observed in the IgAN samples was stronger and more pronounced in comparison to control subjects, however there was no marked difference in the staining intensity in other glomerular diseases group. Therefore, the IHC results confirmed that the three selected proteins appear over-expressed in IgAN vs. healthy controls, but neither CAP1, SHC1 nor PRCP appear significantly upregulated in the kidney tissue of IgAN patients compared to patients with other glomerular diseases. These observations require further investigation in a larger number of samples per CKD aetiology.

**Table 4:** Assessment of IHC staining of the clinical samples for CAP1, SHC1 and PRCP. Intensity of staining in the tissue was graded as “negative/below limit of detection” (−), “weak” (+), “medium” (++) or “strong” (+++). The respective figures are provided in Supplementary Figures 1 and 2. NA – not available.

	Group	Sample ID	Intensity of staining		
			CAP1	SHC1	PRCP
**Cases**	IgAN	305	+++	+++	++
	IgAN	313	+++	+++	++
	IgAN	315	+++	NA	++
	IgAN	312	+++	+++	+++
	IgAN	310	−	+++	+++
	IgAN	302	++	+	+++
	IgAN	306	+	+++	++
	IgAN	307	+	++	++
**Controls**	glomerular disease	311	−	++	+
	glomerular disease	309	+++	+++	+++
	glomerular disease	303	+	++	+
	kidney	kidney	−	+	+

## Discussion

Over the last decades, significant progress was achieved in understanding the pathology of IgAN, however, the critical molecular aspects of the disease remain unexplored^43^. Consequently, current treatment options for IgAN are lacking effectiveness and specificity^14^. In the era of high-throughput omics technologies, integration of heterogeneous data holds the promise of providing new insights, through comprehensive reconstruction and prediction of affected biological processes^44^. Adopting this approach, we integrated urine proteomics datasets mined from the literature, aiming at revealing connections between urinary proteome changes and kidney tissue events, and eventually, discovering novel proteins potentially involved in IgAN pathogenesis. The developed workflow involved extensive data analysis including pathway enrichment followed by a step-wise prioritization of predicted proteins. Taking into account information from high-quality information resources: Nephroseq database for transcriptomics data^45^, Human Protein Atlas for tissue expression data^46^, STRING database for protein-protein interactions^47^, as well as existing knowledge on molecular pathogenesis of IgAN, we obtained a list of candidates meriting further experimental validation and consideration as therapeutic targets. Experimental investigation of three of these shortlisted predicted targets, having IgAN-associated profile and functional relevance, verified their differential expression in the kidney tissue of IgAN patients compared to healthy control samples, further supporting the validity of our approach.

A substantial part of shortlisted proteins obtained from our prediction is involved in pathways related to processes of platelet activation, signalling and aggregation, thus, were pinpointed as potentially important factors of IgAN pathology. Notably, antiplatelet and anticoagulant agents, such as warfarin or statins, have been used for IgAN treatment in Asian countries and evaluated in several controlled studies, but due to poor study design and lack of standardization, definitive conclusions on their efficacy could not be drawn^48, 49^. Platelets are essential for haemostasis and are first responders in vascular injury and endothelial disruption. They are also inflammatory effectors with activities in acute inflammation, but also adaptive immunity and tissue remodelling^50–53^. Platelet activation is common in chronic kidney disease (CKD)^54^, acute kidney injury^55^, and nephrotic syndrome alike^56^. Moreover, platelet degranulation is observed along with atherogenesis in diabetes mellitus, where correlation was found between increased platelet degranulation markers (CD63 and CD40L) and atherosclerosis progression^57^. Proteins found in platelet-related pathways included mediators of inflammatory response such as adenylyl cyclase-associated protein 1 (CAP1), guanine nucleotide-binding protein G(q) subunit alpha (GNAQ) and tyrosine-protein phosphatase non-receptor type 1 (PTPN1), as well as alpha-actinin-1 (ACTN1), alpha-actinin-4 (ACTN4) and growth arrest-specific protein 6 (GAS6), already reported to be differentially expressed in the kidney tissue of IgAN patients. We selected CAP1, a functional receptor of resistin, as a target for validation in the kidney tissue of IgAN patients. Notably, elevated resistin levels in chronic kidney disease have been reported in different studies and are associated with decreased GFR and inflammation^58, 59^. CAP1 is essential for mediation of inflammatory action of monocytes and thus, has been suggested as potential target for treatment of inflammatory diseases^60^. Moreover, induced infiltration of monocytes has been extensively reported in IgAN patients^61, 62^. Given this evidence and the fact that CAP1 has not been studied in the context of kidney disease, it appears to be an excellent candidate molecule for further investigation. The IHC staining confirmed its differential expression in IgAN tissue, in tubules, glomeruli and in inflammatory cells, underlining its potential relevance.

Scavenging of heme from plasma was the second most prominent pathway, identified as upregulated in IgAN. It is related to increased release of haemoglobin (Hb) and heme^63^. Extracellular haemoglobin triggers acute and chronic vascular disease, inflammation, thrombosis, and renal impairment, pathophysiological conditions that are associated with adverse clinical outcomes. Furthermore, oxidative stress, that can be induced by haemoglobin and heme, causes severe damage to tissues - kidney in particular^64^. Along these lines, oxidative stress has been recognized as a mediator of inflammation in IgAN^65, 66^. Moreover, increased levels of plasma Hb and heme may induce platelet activation and thrombosis contributing to vascular inflammation and consequently vascular obstruction^67^. Following our analysis, shortlisted proteins of interest involved in heme scavenging and oxidative stress response included stabilin-1 (STAB1) and SHC-transforming protein 1 (SHC1). STAB1, one of the markers of monocytes, has been found to be associated with cardiovascular diseases^68^. Further studies highlight its role in maintenance of tissue homeostasis and possible therapeutic utility in chronic inflammation^69^. SHC1 is involved in modulating the cellular response to oxidative stress. Drug induced regulation of SHC1 levels may offer a novel therapeutic approach for kidney disease treatment by reducing oxidative stress^70^, a hypothesis which prompted, as a first step, the investigation of SHC1 expression in the IgAN kidney tissue. In support of this hypothesis, a recent study on SHC1 revealed that its isoform p66Shc is a potential novel biomarker of tubular oxidative injury in patients with diabetic nephropathy^71^. Specifically, p66Shc levels were increased in DN patients vs. healthy controls in both, peripheral blood monocytes (PBMs) and renal tissues and positively correlated with the duration of diabetes, levels of triglycerides, HbA1C, LDL-C, blood glucose, tubular interstitial damage and renal oxidative stress. In a mouse model, p66Shc acted as a negative regulator of autoimmune glomerulopathy: p66Shc knockout mice developed a lupus-like autoimmune disease characterized by autoantibody production and immune complex deposition in kidney, resulting in autoimmune glomerulonephritis^72^. These results collectively suggest that overexpression of SHC1, as predicted through our integrative approach and observed following the IHC validation, may be a protective mechanism, meriting further investigation.

Besides the two aforementioned, the metabolism of angiotensinogen to angiotensins was identified in the significantly altered pathways in the analysis. Importantly, polymorphisms in the renin–angiotensin system (RAS) genes have already linked the hypertension aspect of renal diseases to IgAN^73^. Angiotensin II (Ang II) is a well-known cause of glomerular hypertension and hyperfiltration. Moreover, it was reported overexpressed in biopsies of IgAN patients suggesting its stronger impact on IgAN than other glomerular diseases^74^. Similarly, (Pro)renin receptor (PRR) was identified overexpressed in IgAN biopsy samples^40^. PRR was predominantly localized in the cytoplasm of renal tubular cells and its levels correlated with urinary total protein levels, possibly reflecting disease severity^40^. The selected validation candidate - prolylcarboxypeptidase (PRCP), regulates blood pressure and electrolyte balance, cleaving Ang II or Ang III^75^. Along these lines, PRCP deficiency was reported to impair Ang II degradation and thus, its increase may be associated with hypertension and glomerular lesions^76, 77^. PRCP has also been shown to be an activator of the cell matrix-associated pre-kallikrein and molecular events upstream of the fibrinolytic system^78^. In addition, elevated plasma PRCP levels were reported in diabetic and obese patients and significantly increased expression of PRCP during consecutive stages of renal disease development associated with inflammation^79^. In our study, tissue staining showed strong expression of PRCP in kidneys from IgAN patients, with evident, very strong staining in granules. Collectively, these results suggest that PRCP has pleiotropic effects, acting in a context-specific manner, hence further investigation of its role in IgAN appears reasonable.

In summary, although quite simplistic, our analysis captures important aspects of IgAN pathophysiology. We demonstrate the utility of urinary proteomics for biological investigations, and in addition, that data integration provides increased coverage and more insights to processes altered in IgAN, which cannot be predicted by single-set analysis. Several major pathways (and thus, predicted targets), which constitute the backbone of our analysis, were not detected through analysis of each dataset individually or would not appear if the integration step was not applied. Through the application of this systems-level approach to –omics data analysis, we predicted and further experimentally validated key molecules, such as CAP1, SHC1 and PRCP, that might play a significant role in IgAN pathogenesis. Moreover, we highlight other protein targets which could be of interest for further investigation in the context of IgAN.Through the application of this systems-level approach to –omics data analysis, we predicted and further experimentally validated key molecules, such as CAP1, SHC1 and PRCP, that might play a significant role in IgAN pathogenesis. Moreover, we highlight other protein targets which could be of interest for further investigation in the context of IgAN.

Despite being comprehensive, our analysis has several limitations. First of all, we restricted input to urine proteomics datasets that form the majority of published studies for the specific disease. Additionally, given that the extracted datasets were based on comparisons of IgAN vs. healthy controls, the predicted targets might not be specific to IgAN, but reflect general kidney disease. This observation is in part supported by the occasional marked expression of the investigated proteins in kidney tissue with other glomerular diseases (Supplementary Figure 2) and certainly requires further investigation in a larger sample cohort. Furthermore, cofounding factors (for example differences in cohort sizes, patients’ ethnicity, age, sex or comorbidities) are not taken into account, which collectively may affect the analysis output. An additional shortcoming of our study is using transcriptomics datasets and the number of known protein-protein interactions for prioritization of our predictions, through which we shift the emphasis towards known molecular features. However, this is a principal shortcoming of knowledge-based bioinformatics approaches. On a positive note, the obtained enriched pathways reflect better the disease heterogeneity at the level of individual molecules, but also the “common denominator” of the IgAN molecular pathology. Expansion of the analysis to include additional datasets, investigating on this occasion overlaps of IgAN predictions with other autoimmune diseases, in parallel to a more in-depth investigation of the biological relevance and therapeutic potential of the shortlisted targets are warranted.

## Methods

### Preparation of an integrated –omics dataset for the analysis

Proteomics datasets were extracted from the peptide/protein-centric database PeptiCKDdb (www.peptiCKDdb.com)^36^. In brief, peptiCKDdb is a resource of manually curated human proteomics and peptidomics datasets extracted from published scientific studies relative to chronic kidney disease (CKD). The database was searched for datasets extracted from studies related to IgAN, with patients not suffering from IgAN used as controls.

Moreover, PubMed was screened to identify high throughput -omics studies not included in the database. The search (conducted September 2016) included the following keywords: “IgAN AND proteomics” (16 results), “IgAN AND peptidomics” (0 results), “IgAN AND transcriptomics” (1 results), “IgAN AND metabolomics” (1 results), “IgAN AND profiling” (14 results) and was limited to human case-control studies. Obtained references were searched for further relevant datasets of differentially expressed molecules. Datasets consisting of a list of molecules showing differential expression (based on p-value < 0.05 and/or reported criteria) with assigned regulation trend (or fold change/ratio) in IgAN vs. healthy control group were extracted and integrated into a “final” dataset, further used as an input for the pathway enrichment analysis. Molecules appearing in multiple datasets, but showing inconsistent regulation trend were not included in the analysis.

### Pathway enrichment analysis

Pathway enrichment analysis was performed in Cytoscape software (www.cytoscape.org), using ClueGO plug-in for the network visualization. The urine proteomics dataset was introduced as a set of clusters of down- and up-regulated molecules, with the gene name being used as the feature identifier. ClueGO pathway source was set for Reactome pathway database (www.reactome.org). Only statistically significant pathways (p-value < 0.05; two-sided hypergeometric test, Fisher Exact corrected with Bonferroni) were retained. Default parameters were used for Advanced Term/Pathway selection options, Grouping options and CluePedia options. Pathway analysis was performed using two approaches, in order to identify all processes altered in the course of the disease. In the first approach, all extracted features were used as an input for the analysis. In the second approach, major plasma proteins were excluded from the analysis input, based on the hypothesis that their presence is a result of the disease e.g. the disrupted glomerular filtration barrier, rather than a causative event contributing to the development of the disease pathophysiology. To prove the added value of analysis of the integrated dataset vs. single dataset, additional pathway enrichment analysis was performed on each individual dataset and results were evaluated.

### Multi-step finding assessment

Proteins involved in each significant pathway identified in the enrichment analysis, but not being a part of the input dataset, were subjected to multi-step assessment to identify novel (not yet detected in the existing proteomics studies) putative disease-related proteins. Steps followed in the process of shortlisting the predicted molecules are depicted in Fig. 2. Shortlisting was initially performed in an objective, semi-automated manner (Nephroseq concept association analysis with transcriptomics data, described below), followed by a more subjective, final evaluation of potential validation targets based on functional assessment through literature mining.

#### Nephroseq concept association analysis

Concept association analysis is available from the Nephroseq database (www.nephroseq.org, version September 2016)^45^. Nephroseq concepts are groups of differentially expressed genes in specific diseases. They are derived from deposited datasets that involve comparison of at least two groups. Each concept contains top 1, 5, and 10 percent of the over- and under-expressed genes from the respective dataset. Concept association analysis allows for comparison of user gene/protein dataset to genes in Nephroseq-derived concepts, in order to identify overlapping molecular features. The list of proteins predicted in pathway analysis of the urine proteomics datasets was used as input in Nephroseq concept association analysis. Default analysis settings were applied: odds ratio threshold = 2, p-value threshold = 0.0001. Significant concepts (p-value < 0.05, having at least 3 overlapping genes with the input data) related to IgAN were retained. The respective demographics, pathology or tissue type are summarized in Supplementary Table S6. Molecules overlapping between the input proteomic dataset and Nephroseq concepts related to IgAN were selected for further evaluation.

#### Tissue expression, PPIs, occurrence in pathways

The molecules highlighted from the concept association analysis were investigated for expression in healthy kidney tissue through the Human Protein Atlas (www.proteinatlas.org)^46^. Features with IHC evidence for kidney expression were retained for further investigation. Similarly, for each protein, the number of pertinent protein-protein interactions in the corresponding pathway was estimated (STRING database^47^; www.string-db.org; prediction methods: all, score: highest confidence (0.9)). Additionally, the frequency of occurrence of each molecule in the identified pathways was calculated. Of note, pathways sharing the same parent node (e.g. platelet activation, signalling and aggregation is the parent node for platelet degranulation and signal amplification pathways) were combined and considered as one pathway. Molecules being involved in > 1 PPI and/or present in > 1 pathway were subjected to further functional assessment.

#### Functional assessment

Initial functional evaluation was performed using UniProt (www.uniprot.org) and GeneOntology (www.geneontology.org) databases, with focus on protein function and relevant biological processes. Given the pathological background of IgAN, involvement in immunity/autoimmunity, blood pressure regulation, vascular injury, oxidative stress and ECM remodelling was considered for protein shortlisting. Subsequently, shortlisted molecules were searched in the literature resources (PubMed/ Web of Science) to further shortlist proteins meriting further experimental verification.

### Experimental validation

#### Human kidney biopsy samples

Sections of paraffin-embedded human kidney tissue were obtained from biopsy-proven IgAN patients (n = 8) and patients with other glomerular diseases (tubulointerstitial nephritis, focal segmental glomerulosclerosis and mesangial proliferative glomerulonephritis, n = 3). Normal kidney control tissue was obtained from the normal kidney region after renal tumour nephrectomy. Clinical characteristics of patients are included in the Supplementary Table S10. Sample collection was performed in accordance to local ethics requirements and the study was approved by the local ethics committees of Wroclaw Medical University, Wroclaw, Poland (No. KB-88/2013), Timisoara County Emergency Clinical Hospital, Timisoara, Romania (No. 84; 8/08/2015) and University of Glasgow. The “CKD-BIO Study” was approved by the institutional review board of the Ethic Subcommittee for Medicine, Pharmacy, Veterinary and Stomatology of the Macedonian Academy of science and Arts (12/02/2015). All individuals gave written informed consent. Institutional review board approval was obtained for procurement of kidney specimens at the University of Glasgow.

#### Immunohistochemistry

Immunostaining of paraffin-embedded kidney tissues was performed on 2.5-µm tissue sample sections using UltraVision Quanto Detection System (Thermo Scientific), following the manufacturer’s instructions. Primary antibodies included rabbit recombinant monoclonal anti-CAP1, rabbit recombinant monoclonal anti-SHC and rabbit polyclonal anti-PRCP antibodies provided by Abcam (ab155079, ab33770, ab171846 respectively). Incubation with primary antibody was performed with the following dilutions: CAP1 (1:100), SHC1 (1:400), PRCP (1:20). After counterstaining with haematoxylin, slides were dehydrated and mounted with DPX mounting medium. Slides were digitized using Hamamatsu NDP slide scanner and viewed on Slidepath Digital Image Hub (Leica Microsystems). Intensity of staining in the tissue was graded in different kidney compartments (tubules, endothelial cells, glomeruli) as “negative” (−), “weak” (+), “medium” (++) or “strong” (+++). For each staining target, negative control was performed through omission of primary antibody.

### Data availability

All data generated or analysed during this study are included in this published article (and its Supplementary Information files).

## Electronic supplementary material

Supplementary Information

Supplementary Datasets
