# Supplementary material for: Identification of novel molecular signatures of IgA nephropathy through an integrative -omics analysis
Source: Sci Rep. 2017 Aug 22;7:9091. doi: 10.1038/s41598-017-09393-w (PMC5567309; doi:10.1038/s41598-017-09393-w)
Supplement: Supplementary file 1 — Supplementary Information [file 41598_2017_9393_MOESM1_ESM.pdf]

Identification of novel molecular signatures of IgA nephropathy through an integrative -omics analysis

Supplementary Information

Magdalena Krochmal[1,2], Katryna Cisek[3], Szymon Filip[1], Katerina Markoska[9], Clare Orange[8], Jerome Zoidakis[1], Chara Gakiopoulou[10], Goce Spasovski[9], Harald Mischak[3,6], Christian Delles[7], Antonia Vlahou[1*], Joachim Jankowski[2,5*]

[1]Biomedical Research Foundation Academy of Athens, Center of Basic Research, Athens, Greece,

[2]RWTH Aachen University Hospital, Institute for Molecular Cardiovascular Research, Aachen, Germany,

[3]Mosaiques Diagnostics GmbH, Mosaiques Diagnostics GmbH, Hannover, Germany,

[5]University of Maastricht, CARIM School for Cardiovascular Diseases, Maastricht, Netherlands,

[6]University of Glasgow, Institute of Cardiovascular and Medical Sciences, Glasgow, UK

[7]Institute of Cardiovascular and Medical Sciences, BHF Glasgow Cardiovascular Research Centre, University of Glasgow, 126 University Place, Glasgow, G12 8TA, UK

[8]Department of Pathology, School of Medicine, University of Glasgow, Glasgow, UK.

[9]Department of Nephrology, Medical Faculty, University of Skopje, Skopje, Macedonia

[10]Pathology Department, National and Kapodistrian University of Athens, Athens, Greece

Supplementary table and figure legends

Supplementary figures

Supplementary Figure S1. Immunohistochemical staining of A) CAP1, B) SHC1, C) PRCP in IgAN Samples, Scale bar - 100 μm. Sample information available in Supplementary Table S10.

Supplementary Figure S2. Immunohistochemical staining of A) CAP1, B) SHC1, C) PRCP in control samples of other glomerular diseases and healthy kidney, Scale bar - 100 μm. Sample information available in Supplementary Table S10.

Supplementary Tables (Supplementary_data.xls)

Supplementary Table S1. Supplementary Table S1. List of proteins extracted from 9 urinary proteomics manuscripts and used for data integration. Proteins CO6A1_HUMAN, CYTC_HUMAN, DPP4_HUMAN, UROM_HUMAN were excluded from the analysis due to inconsistent regulation among datasets.

Supplementary Table S2. List of 232 proteins obtained after data pre-processing and integration step, used as an input for pathway analysis (Cytoscape, CLUEGO) in Approach 1 (see Methods section).

Supplementary Table S3. List of 190 proteins obtained after data pre-processing and integration step, followed by exclusion of bona fide plasma proteins, used as an input for pathway analysis (Cytoscape, CLUEGO) in Approach 2 (see Methods section).

Supplementary Table S4. List of biological pathways yielded in pathway enrichment analysis of integrated dataset of 232 proteins (Approach 1, full protein list). Pathway names marked grey were excluded from analysis as insignificant in the context of the study.

Supplementary Table S5. List of biological pathways yielded in pathway enrichment analysis of 190 proteins from the integrated dataset (Approach 2, after plasma proteins exclusion). Pathway names marked grey were excluded from analysis as insignificant in the context of the study.

Supplementary Table S6. Nephroseq concept association analysis results. IgAN-related concepts and 223 corresponding genes overlapping with the analysis input data (657 pathway building proteins).

Supplementary Table S7. List of 223 proteins remaining after concept association analysis ("Nephroseq" database). Each protein is annotated with number of predicted pathways in which it

appears, number of formed PPIs (“STRING” database), kidney tissue expression (“Human Protein Atlas”) and relevance of function (in the context of IgAN).

Supplementary Table S8. List of 68 proteins remaining after multi-step assessment.

Supplementary Table S9. Comparison of pathways yielded through analysis of integrated dataset and each dataset individually. Pathways marked yellow were considered significant and found only after datasets integration.

Supplementary Table S10. Clinical sample information and IHC staining assessment. Intensity of staining in the tissue was graded as “negative/below limit of detection”(−), “weak”(+), “medium”(++) or “strong”(+++).

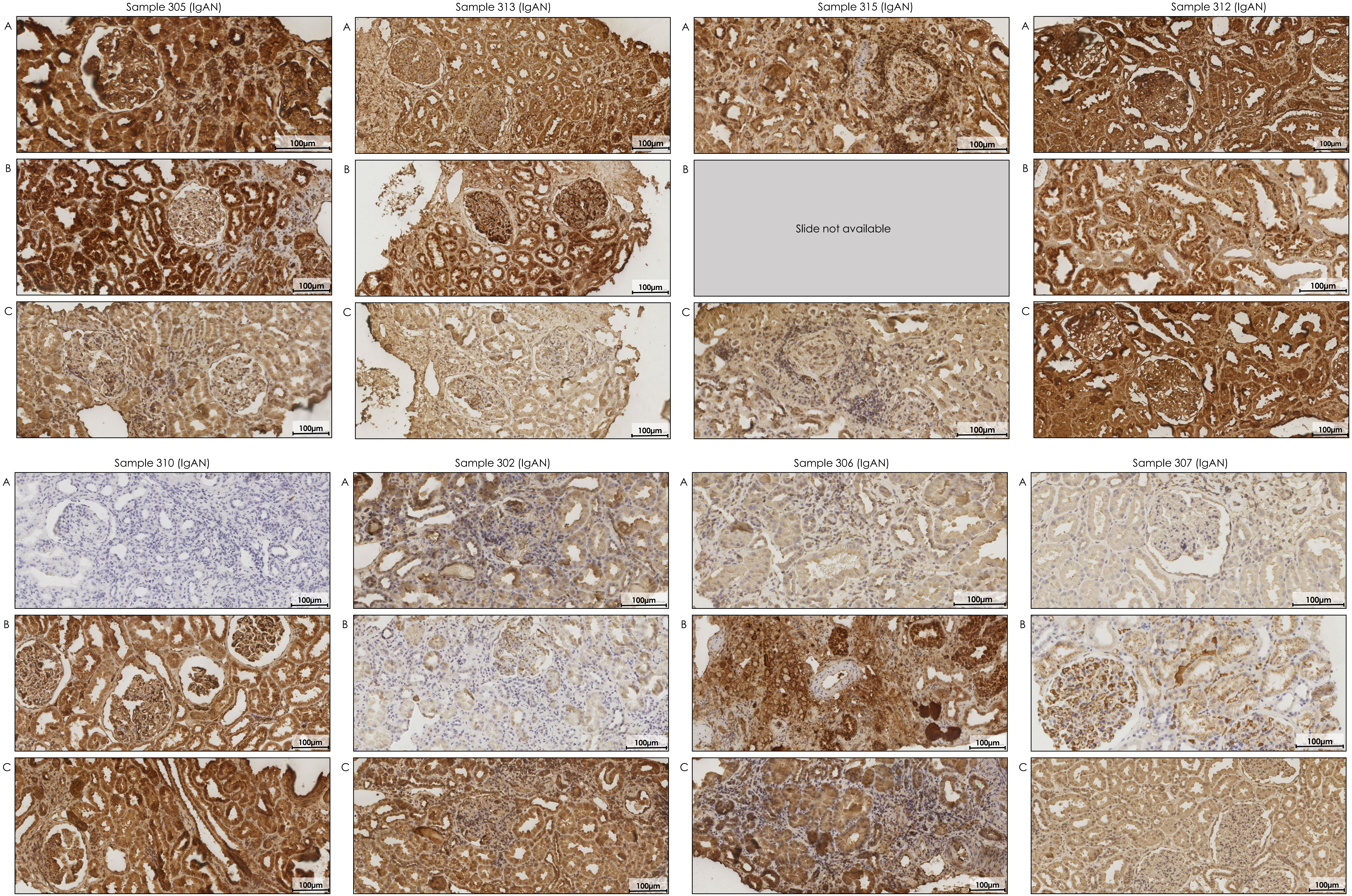

Supplementary Figure S1. Immunohistochemical staining of A) CAP1, B) SHC1, C) PRCP in IgAN Samples, Scale bar - 100 µm. Sample information available in Supplementary Table S10.

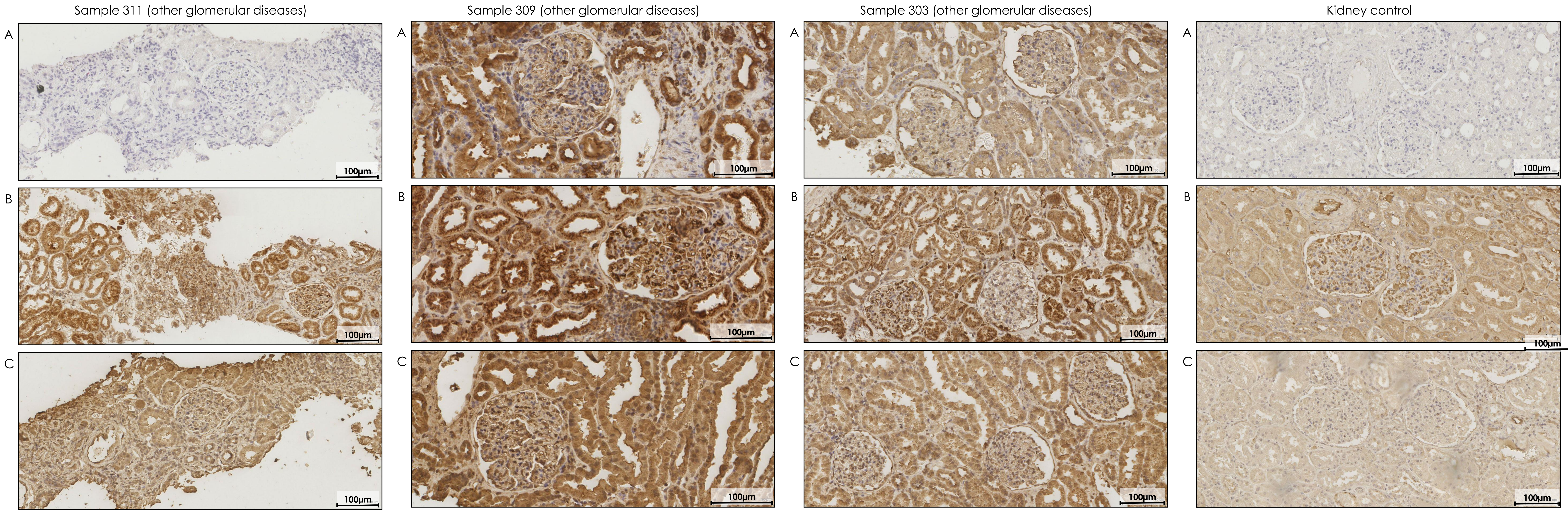

Supplementary Figure S2. Immunohistochemical staining of A) CAP1, B) SHC1, C) PRCP in control samples of other glomerular diseases and healthy kidney, Scale bar - 100 μm.

Sample information available in Supplementary Table S10.
